# Topical sulforaphane exacerbates disease and promotes the accumulation of inflammatory CD8+ T cells in a dermatitis model

**DOI:** 10.64898/2026.09.11.751007

**Published:** 2026-09-18

**Authors:** Anthony Tukanowicz-Hassett, Sagar Pradeep Bapat, Yuichi Yokoyama, Taku Kambayashi

## Abstract

Background: Atopic dermatitis (AD) is a chronic inflammatory skin disease characterized by skin barrier dysfunction and immune activation. Antioxidants such as sulforaphane (SFN) have shown benefit in mouse models of AD and are being explored as topical therapies. Objectives: We investigated the immune mechanisms through which topical SFN affects skin pathology in the MC903-induced AD-like dermatitis. Materials and Methods: We used flow cytometry to characterize skin immune cell infiltration, activation, and cytokine production. Genetic and pharmacological loss-of-function studies were used to determine the contribution of specific immune cell populations and signaling pathways. Results: Although topical application of SFN by itself caused no skin inflammation, it exacerbated MC903-induced dermatitis. Disease exacerbation was accompanied by robust T cell infiltration to skin, particularly by CD8 + T cells, and was significantly attenuated in TCRβ-deficient and β2 m-deficient mice. In addition to a Type 2 inflammatory response, the skin CD8 + T cells exhibited a Type 1 inflammatory program characterized by IFNγ production that was largely absent among CD4 + T cells. Loss of IFNγ signaling attenuated SFN-mediated disease exacerbation. Blocking lymphocyte egress with FTY720 markedly reduced the SFN-induced increase in skin CD8 + T cells, suggesting that the increase was primarily dependent on recruitment from the periphery rather than local expansion. Conclusion: Topical SFN worsens MC903-induced dermatitis through a mechanism involving the peripheral accumulation of IFNγ-producing CD8 + T cells in skin. These findings demonstrate that SFN can exert context-dependent pro-inflammatory effects and underscore the need to evaluate its effects carefully when considering topical medical or cosmetic applications.

## Full Text Availability

The license terms selected by the author(s) for this preprint version do not permit archiving in PMC. The full text is available from the preprint server.

